# The Essentials of Medical Chemistry and Urinalysis

**Published:** 1890-12

**Authors:** 


THE ESSENTIALS OF MEDICAL CHEMISTRY AND URINALYSIS. By SAM. E. WOODY, A. M., M. D., Professor of Chemistry and Public Hygiene, and Clinical Lecturer on Diseases of Children in the Kentucky School of Medicine. Third edition; revised, enlarged and illustrated; pp. 157. P. Blakiston, Son & Co., Philadelphia, 1890.

Upon reading the title page of this little work we hoped to find a brief outline of the chemistry of the human body in its relation to physiology and pathology. But alas for human hopes! A half-dozen pages had not been read before our hopes and anticipations vanished like mist before the noon-day sun.

If teachers of medical chemistry would only step out of the rut they have been traveling for years and teach the subject as it should be, physicians would appreciate this branch of the science far more than they do, and chemistry, instead of being considered of only passing importance, would soon be elevated to its proper sphere. We realize that many institutions have too brief a course, in which many subjects of direct importance must be handled, to give the student more than a passing outline of chemistry. Under such conditions we would advise that the course be lengthened, and the first step be taken to fall in line with those colleges that are endeavoring to advance medical education; that the student be first thoroughly grounded in the principles of chemistry, and that medical chemistry be then taught from the vantage ground of physiology and pathology. Until this is done, the average student will derive but little benefit from his study of chemistry at college. He sees, in the "atomic theory," the "laws of definite and multiple proportions," or even in the properties of the elements and their compounds, no direct bearing upon or connection with medicine, and is liable to do only as much as is required to pass his examination, and six months after this is passed he knows so little that his teacher blushes when he realizes the fact.

Of this little work before us we can only say that it is not what it is claimed to be. It is nothing more than a brief outline of general chemistry, with a chapter on the urine and its examination.

An examination of the text shows a lack of clearness, which is so essential to the student.

We are glad that the author has discarded the old obsolete classification of the elements into metals and non-metals.

Under the chapter Water, the author says:

> Though chemical analysis cannot detect the disease-producing elements, it can detect organic impurity, without which they cannot exist This is easily done thus: (1) Half fill a clean bottle with the water, warm, agitate and critically smell it. A foul odor indicates organic impurity. (2) Fill a clean pint bottle three-fourths full, add a teaspoonful of the purest white sugar or gelatine; set aside, in a warm place, for two days, when, if it becomes cloudy (bacteria), it is unfit for use.

While these methods are, as the doctor says, only "rough-and-ready tests," they are very liable to lead the physician or his patient into a false feeling of security. The only proper way to determine the organic purity of a potable water is by chemical analysis, and this should be done by one trained for such work, and who is competent to interpret correctly the results obtained.

The statement made that aqua-regia is the only solvent of gold and platinum, is erroneous.

In the portion of the work treating on organic chemistry under the head of Ethers, the author says (after describing the process of manufacture): "The sulphuric acid is said to act by its mere presence, by *catalysis;* or in other words, it acts because it acts." . . . . We consider the manner in which this statement is put as entirely unwarranted in the light of modern investigation. Had the author said: "The action of the sulphuric acid was formerly explained by catalysis," and then gone on to say that Williamson had proven the true rationale to be as follows (giving it), no one would be liable to suppose that the professor was expounding some new unheard-of explanation. The author, in our opinion, does not give those who have gone before him sufficient credit for the work done.

While this little book is printed in the first-class style so characteristic of the publishers, there are a number of typographical errors which should not be in a third edition. J. A. M.

